# Estimating the dimensionality of the manifold underlying multi-electrode neural recordings

**DOI:** 10.1371/journal.pcbi.1008591

**Published:** 2021-11-29

**Authors:** Ege Altan, Sara A. Solla, Lee E. Miller, Eric J. Perreault

**Affiliations:** 1 Department of Neuroscience, Northwestern University, Chicago, Illinois, United States of America; 2 Department of Biomedical Engineering, Northwestern University, Evanston, Illinois, United States of America; 3 Department of Physics and Astronomy, Northwestern University, Evanston, Illinois, United States of America; 4 Department of Physical Medicine and Rehabilitation, Northwestern University, Chicago, Illinois, United States of America; 5 Shirley Ryan AbilityLab, Chicago, Illinois, United States of America; Dartmouth College, UNITED STATES

## Abstract

It is generally accepted that the number of neurons in a given brain area far exceeds the number of neurons needed to carry any specific function controlled by that area. For example, motor areas of the human brain contain tens of millions of neurons that control the activation of tens or at most hundreds of muscles. This massive redundancy implies the covariation of many neurons, which constrains the population activity to a low-dimensional manifold within the space of all possible patterns of neural activity. To gain a conceptual understanding of the complexity of the neural activity within a manifold, it is useful to estimate its dimensionality, which quantifies the number of degrees of freedom required to describe the observed population activity without significant information loss. While there are many algorithms for dimensionality estimation, we do not know which are well suited for analyzing neural activity. The objective of this study was to evaluate the efficacy of several representative algorithms for estimating the dimensionality of linearly and nonlinearly embedded data. We generated synthetic neural recordings with known intrinsic dimensionality and used them to test the algorithms’ accuracy and robustness. We emulated some of the important challenges associated with experimental data by adding noise, altering the nature of the embedding of the low-dimensional manifold within the high-dimensional recordings, varying the dimensionality of the manifold, and limiting the amount of available data. We demonstrated that linear algorithms overestimate the dimensionality of nonlinear, noise-free data. In cases of high noise, most algorithms overestimated the dimensionality. We thus developed a denoising algorithm based on deep learning, the “Joint Autoencoder”, which significantly improved subsequent dimensionality estimation. Critically, we found that all algorithms failed when the intrinsic dimensionality was high (above 20) or when the amount of data used for estimation was low. Based on the challenges we observed, we formulated a pipeline for estimating the dimensionality of experimental neural data.

## Author summary

The number of neurons that we can simultaneously record from has increased exponentially for decades; today that number is in the thousands of neurons. However, the individual firing rates are highly redundant. One approach to identifying important features from redundant data is to estimate the dimensionality of the neural recordings, which represents the number of degrees of freedom required to describe the data without significant information loss. Better understanding of dimensionality may also uncover the mechanisms of computation within a neural circuit. Circuits carrying out complex computations might be higher-dimensional than those carrying out simpler computations. Typically, studies have quantified neural dimensionality using one of several available methods reflecting a lack of consensus on which method would be most appropriate for neural data. In this work, we used several methods to investigate the accuracy of simulated neural data with properties mimicking those of actual neural recordings. Based on these results, we devised an analysis pipeline to estimate the dimensionality of neural recordings. Our work will allow scientists to extract informative features from a large number of highly redundant neurons, as well as quantify the complexity of information encoded by these neurons.

## Introduction

Studies that simultaneously record the activity of many neurons have shown that cortical neural activity is highly redundant [1]. In primary motor cortex (M1), redundancy arises as tens of millions of neurons control tens or at most hundreds of muscles. This redundancy implies significant covariation in the activity of many neurons, which confines the population neural activity to a low-dimensional manifold embedded in the neural space of all possible patterns of neural population activity [2–9]. Low-dimensional manifolds have also been observed in a variety of other cortical regions [10–18]. Reliable algorithms for identifying these manifolds and characterizing their dimensionality are increasingly important as our ability to record from large populations of neurons increases [19]. The dimensionality of the manifold describing the coordinated firing of a set of neurons quantifies the number of degrees of freedom needed to describe population activity without significant information loss [20,21]. Projecting the observed firing patterns onto the manifold yields a low-dimensional set of latent signals that can simplify the interpretation of population neural activity [2,9,22]. Low-dimensional latent signals can facilitate the manipulation or the extraction of signals for brain-computer interfaces, a rehabilitative technology that converts neural signals into control commands to restore movement to paralyzed patients [23,24].

Unfortunately, it is surprisingly difficult to estimate the dimensionality of neural manifolds, particularly in the realistic condition of a noisy, nonlinear embedding. There is evidence of a nonlinear mapping between the recorded neural activity and the associated low-dimensional latent signals [10,25–27]. Noise propagates from the level of sensory transduction and amplification, the opening and closing of voltage-gated ion channels, and builds up at the level of synapses, causing neural firing to be a stochastic process [28]. The two effects, nonlinearity and noise, combine to pose significant challenges to existing dimensionality estimation algorithms. The accuracy of the estimators also depends on the amount of available data [29,30], which is limited in most experimental paradigms. If we wish to identify the manifolds associated with experimentally measured neural activity, we need methods that are robust in the presence of these challenges.

The methods that have been proposed for estimating the dimensionality of neural manifolds can be broadly categorized into linear or nonlinear algorithms, based on assumptions about the nature of the mapping between the low-dimensional representation of the latent signals and the high-dimensional space of neural activity. The most commonly used linear method for dimensionality reduction is Principal Component Analysis (PCA), based on identifying mutually orthogonal directions in the empirical neural space of recorded activity; these directions are monotonically associated with the largest data variance. PCA provides a hierarchical description in which the data projected onto the manifold subtended by the leading principal components become closer and closer to the recorded data as the dimensionality of the linear manifold is increased towards the dimensionality of the empirical neural space. Although PCA provides a useful and systematic tool for variance-based dimensionality reduction, it does not specify how to uniquely identify the dimensionality of the manifold: the typical implementation requires the choice of an arbitrary variance threshold. Other PCA-based algorithms such as Participation Ratio (PR) [5,18] and Parallel Analysis (PA) [31,32] provide more principled prescriptions for linear dimensionality estimation, by incorporating criteria for determining an optimal number of leading principal components to use when constructing the low-dimensional manifold.

Linear dimensionality estimation algorithms may work well for linear datasets, but are likely to overestimate the dimensionality of a manifold arising from a nonlinear mapping between the low-and high-dimensional spaces [20,21,33,34]. In contrast, nonlinear methods (e.g., Correlation Dimension [35–37], Levina-Bickel Maximum Likelihood Estimation [38], Two Nearest Neighbors [39], and Fisher Separability Analysis [40]) may provide accurate dimensionality estimates for both linearly and nonlinearly embedded data.

Most dimensionality estimation methods have been tested in the absence of noise even though it is known that linear and nonlinear methods overestimate dimensionality when the data is noisy [20]. The robustness of dimensionality estimation algorithms to noise remains to be characterized.

The objective of this study was to characterize the accuracy of several dimensionality estimation algorithms when applied to high-dimensional recordings of neural activity. We evaluated previously proposed algorithms on synthetic datasets of known dimensionality to identify conditions under which each method succeeded and/or failed. Specifically, we evaluated how the algorithms handled the nature of the embedding (linear or nonlinear), the amount of noise added to the simulated neural data, and the amount of data available. We found increasing levels of noise to be a challenge for all tested algorithms. We therefore also evaluated different approaches for reducing noise prior to performing dimensionality estimation, including the “Joint Autoencoder”, a method we developed based on deep learning techniques. Together, our results allowed us to propose a methodological pipeline for estimating the intrinsic dimensionality of high-dimensional datasets of recorded neural activity.

## Methods

### Ethics statement

All surgical and experimental procedures that yielded the multi-electrode array recordings from non-human primates [41], which formed the basis of our simulated neural signals, were approved by Institutional Animal Care and Use Committee (IACUC) of Northwestern University. The subject was monitored daily. The subject’s diet consisted of standard laboratory animal diet, fresh fruits, and vegetables, and was provided with access to various types of enrichment.

### Simulation of neural signals

We generated the synthetic data used to evaluate the various dimensionality estimation algorithms as follows. First, we created *d* signals by randomly selecting (*d* x *M*) samples from an empirical distribution of firing rates that we obtained from multi-electrode array recordings of neural activity in the macaque primary motor cortex (M1) made while the monkey was performing a center-out task [41]. The firing rates were binned at 50 ms. The sampling was done randomly across all recorded neurons and time bins within successful trials. Our goal was to generate *M* samples of *d*-dimensional latent variables that were uncorrelated with each other and individually uncorrelated over time; we verified that these randomly selected signals were indeed uncorrelated, as intended. These signals provided a set of variables of known dimension *d* that preserved the first-order firing statistics of the neural activity recorded in M1. Our procedure aimed at generating simulated data that reproduces possible states of activity of a neural population, without considering the order in which these states might be visited; in other words, we focused on population statistics as opposed to population dynamics.

**Fig 1:**
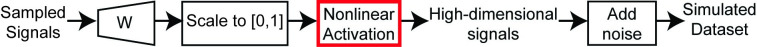
Generation of simulated datasets. First, latent neural signals were obtained by randomly sampling the firing rates of primary motor cortical recordings. The number of latent signals determined the intrinsic dimensionality of the dataset. Then, the dimensionality of the dataset was increased through linear combinations effected by multiplication with a weight matrix *W*. The entries of *W* were sampled from a zero-mean Gaussian distribution with unit variance. The resulting signals were then scaled to the [0,1] range by dividing them by their maximum value. This procedure yielded noise-free, linear datasets. In nonlinear simulations, the signals were activated nonlinearly using the exponential function in Eq 1 (red box in diagram). In noisy simulations, zero-mean Gaussian noise with variance specified by a predetermined signal-to-noise ratio was added to the signals. This procedure yielded linear or nonlinear, noisy datasets with known signal-to-noise ratio.

These signals provided a *d*-dimensional latent set used to construct synthetic high-dimensional data sets **(Fig 1)**. We allowed *d* to vary from 3 to 40. For the analyses where a fixed value of *d* was used we chose *d* = 6, to approximate the characteristics of real data collected in our laboratory (**S1 Table)**. The *d*-dimensional latent signals were first smoothed using a Gaussian kernel (s.d.: 50 ms), and then multiplied by a *N* x *d* mixing matrix *W* with entries that were randomly selected from a zero-mean Gaussian distribution with unit variance. This resulted in a dataset *X* composed of *M* samples, each of them *N*-dimensional. We chose *N* = 96 to reproduce the number of signals recorded by the multi-electrode array used to obtain the original experimental data. The activity in each of the *N* = 96 simulated channels was scaled to the range from zero to one to compensate for variability in firing rates across neurons and across time. The effect of non-uniform firing rate variances across channels was considered separately (see “Effect of non-uniform variances across channels” in Results).

A nonlinear embedding was implemented by processing each simulated neural recording in *X* with an exponential activation function:

$$f(X)=\frac{e^{\alpha X}-1}{e^{\alpha }-1}$$ (Eq 1)

The choice of an exponential nonlinearity was based on results from Generalized Linear Models, for which the statistics of the modeled variable determines the nonlinear link function [42]. In our case, the variables of interest are spike counts. Under the assumption that these variables follow a Poisson-like distribution, the appropriate choice of link function is the logarithm [42]. The inverse of the link function, the exponential, is the appropriate nonlinear function for relating the linear combination of explanatory covariates, the latent signals, to the variables of interest, the simulated firing rates. The exponential activation function used in our simulations allowed us to control the degree of nonlinearity by varying the single parameter α, and to ensure that the range of the nonlinearly embedded synthetic data remained between zero and one. Finally, we added independent Gaussian noise to each of the channels in *X*, to generate signals with known signal-to-noise ratio. This choice of noise model provides a simple and widely used mechanism for simulating stochastic processes [43].

The various steps in this procedure allowed us to generate datasets of known intrinsic dimensionality, embedding type (linear/nonlinear), and signal-to-noise ratio.

### Dimensionality estimation algorithms

We evaluated two classes of dimensionality estimation algorithms, those that assumed a linear embedding and those that also allowed for a nonlinear embedding.

#### Linear algorithms

Linear algorithms map high-dimensional data to a lower dimensional, linear subspace. Principal Component Analysis (PCA) is often used for linear dimensionality estimation in neuroscience [2,4,7,41,44,45]. All the linear algorithms that we tested (summarized below) are based on PCA but use different criteria for dimensionality estimation.

#### Principal Component Analysis with a variance cutoff

PCA creates a low-dimensional representation of the data by sequentially finding orthogonal directions that explain the most remaining variance. Unit vectors that identify those directions, the PCA eigenvectors {*v*_*i*_}, provide an orthonormal basis for the *N*-dimensional data space. The eigenvectors are labeled in decreasing order of the variance associated with each direction, given by the eigenvalues {*λ*_*i*_}. The simplest way to use PCA for dimensionality estimation is to find the number of principal components required to reach a predetermined threshold of cumulative variance. The selection of a variance threshold can be rather arbitrary, and a range of thresholds have been used in the literature. In this study, we used a threshold of 90%, which yielded accurate estimates of dimensionality for the noise-free linear datasets.

#### Participation Ratio (PR)

This approach provides a principled way of finding a variance threshold when the ground truth is not known [5,18]. PR uses a simple formula based on the eigenvalues:

$$PR=\frac{{\left(\sum_{i=1}^{N}\lambda_{i}\right)}^{2}}{\sum_{i=1}^{N}{\left(\lambda_{i}\right)}^{2}}$$ (Eq 2)

If the leading eigenvalue carries all the variance (*λ*_*i*_ ≠ 0 for *i* = *1* and *λ*_*i*_ = 0 for all *i* ≥ 2), then PR = 1. At the other extreme, if all eigenvalues are equal, the variance is spread evenly across all the dimensions, and PR = *N*. The actual value of PR interpolates between these two extreme conditions to estimate the intrinsic dimensionality, and thus the number of principal components to be kept [5].

#### Parallel Analysis (PA)

Much like the Participation Ratio, Parallel Analysis is a principled approach to finding a variance threshold [31,32]. Parallel Analysis generates null distributions for the eigenvalues by repeatedly shuffling each of the *N* dimensions of the data separately. The shuffling step ensures that the remaining correlations across the different dimensions of the data are due to chance. We repeated the shuffling procedure 200 times, resulting in a null distribution for each eigenvalue based on 200 samples. The eigenvalues that exceeded the 95^th^ percentile of their null distribution were identified as significant; the number of significant eigenvalues determined the number of dimensions to be kept. Although this method has not been directly applied to neural data, similar approaches based on finding null distributions of eigenvalues have been used for neural dimensionality estimation [46].

#### Nonlinear algorithms

Nonlinear algorithms can in principle estimate the dimensionality of either linearly or nonlinearly embedded data. Unlike the linear algorithms we tested, the nonlinear algorithms need not rely on a global model for the probability distribution from which the data are assumed to be drawn (in the case of PCA, the model is a multivariate Gaussian distribution). Instead, many nonlinear algorithms estimate intrinsic dimensionality directly from local geometric properties of the data. Commonly used local properties include distance and separability of each data point relative to its neighbors. Although nonlinear algorithms are not yet much used in neuroscience, they have been used to estimate dimensionality in several other fields that produce high-dimensional datasets [47].

#### Correlation Dimension (CD)

Correlation Dimension estimates dimensionality by calculating how the number of data samples that fall within a hypersphere change as a function of its radius. This method, originally developed in 1983 [35], has benefitted from recent efforts to improve computational speed and accuracy [36,37]. Although there are only a few applications of Correlation Dimension analysis to neural data [48,49], it is widely used in other disciplines [36].

#### Levina-Bickel Maximum Likelihood Estimation (LBMLE)

The Levina-Bickel Maximum Likelihood Estimation method [38] is an extension of Correlation Dimension that uses a maximum likelihood approach to estimate distances between data points. This method has been successfully applied to some of the benchmark datasets used in machine learning, such as the Faces [33] and Hands datasets [50].

#### Two Nearest Neighbors (TNN)

The Two Nearest Neighbors method also uses the distance between data points to estimate dimensionality [39]. However, unlike Levina-Bickel Maximum Likelihood Estimation, it considers only the first and second neighbors of each point. The ratio of the cumulative distribution of second-neighbor to first-neighbor distances is a function of data dimensionality. By focusing on shorter distances, the method avoids unwanted effects resulting from density changes across the manifold. This method has been successfully applied to synthetic datasets of hyperspheres with known dimensionality [39], and to real-world datasets including molecular simulations [51] and images of hand-written digits [33].

#### Fisher Separability Analysis (FSA)

High-dimensional datasets exhibit simple geometric properties such as the likely orthogonality of two randomly picked directions. These properties have recently been characterized as the *blessings of dimensionality* [52], in contrast to the well-known concept of the *curse of dimensionality*. A useful example is the increasing ease with which a hyperplane can separate any given sample in a dataset from all other samples as the dimensionality of the dataset increases. Fisher separability is a computationally efficient, simple, and robust method to assess such separability [53,54]. Dimensionality can be estimated in terms of the probability that a point in the dataset is Fisher separable from the remaining points [40]. The probability distribution of Fisher separability allows the dimensionality of both linear and nonlinear manifolds to be estimated. This method has been applied to study the mutation profiles of the genes resulting in tumors as a means to evaluate therapeutic approaches [55].

### Denoising algorithms

Noise that is uncorrelated across channels will lead to dimensionality estimates that approach the number of channels as the level of noise increases. To mitigate this overestimation problem, we implemented two approaches to denoise neural data. Both rely on an initial estimate of an upper bound dimensionality *D*, estimated here by using Parallel Analysis. To quantify the performance of the denoising algorithms, we reported Variance Accounted For (VAF) between the denoised signals and the noise-free signals, the latter providing the ground truth.

#### PCA denoising

The linear approach to denoising was based on PCA. Once the value of *D* was determined, we used the *D* leading principal components to reconstruct the original data. PCA-based denoising is based on the assumption that most of the noise is relegated to the discarded, low-variance principal components.

#### Joint Autoencoder denoising

**Fig 2:**
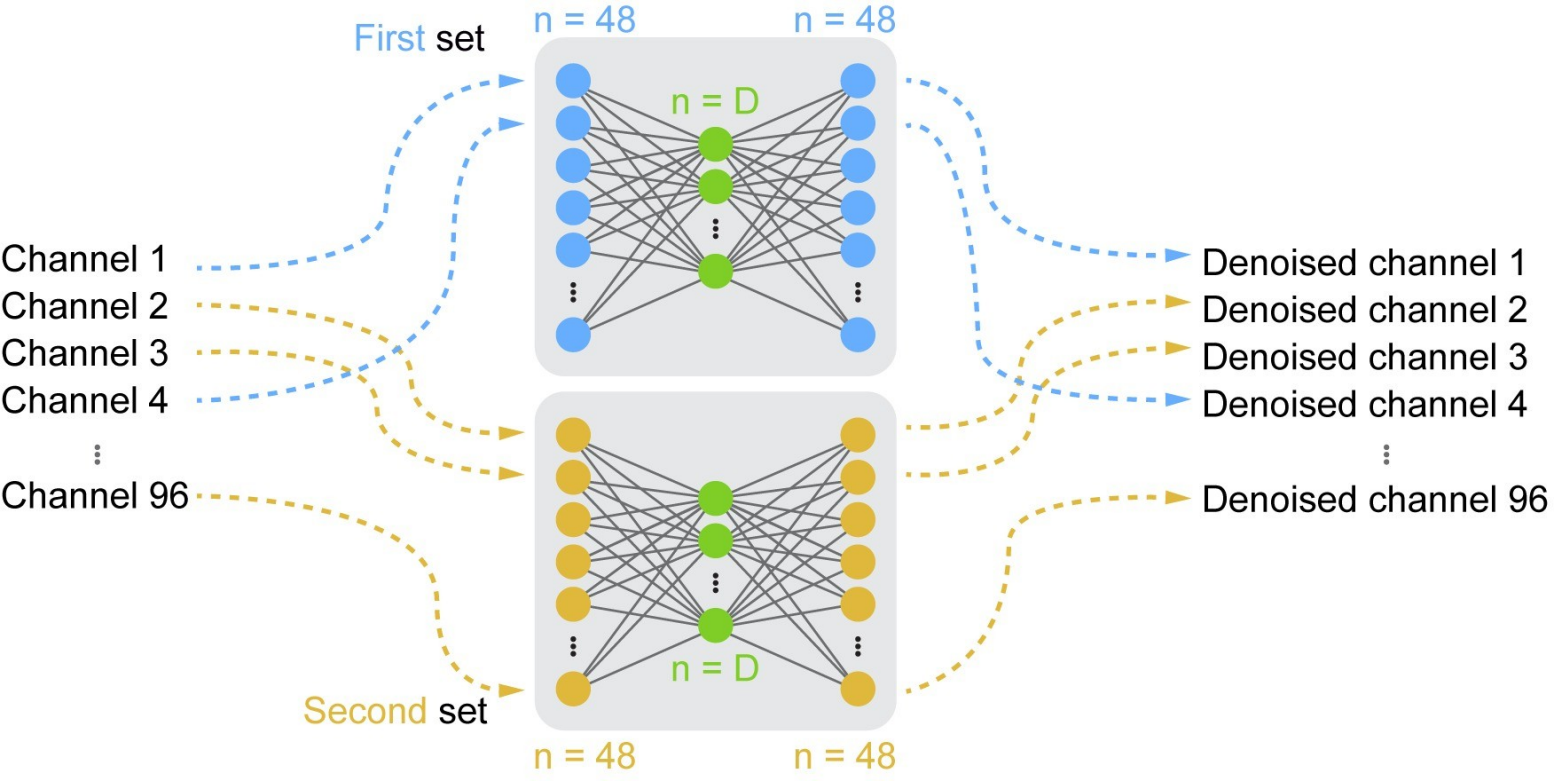
Architecture of the Joint Autoencoder. Channels of the 96-dimensional simulated datasets were randomly partitioned into two sets of signals (blue and yellow). Each 48-dimensional set was reconstructed through the corresponding *D*-dimensional subspace, *Z*_1_ and *Z*_2_ (green). The reconstructed outputs of the networks were the denoised channels.

We also used a neural network for denoising **(Fig 2**). For this purpose, we divided the 96-dimensional simulated dataset *X* into two 48-dimensional partitions: *X*_1_ and *X*_2_. These partitions were each mapped by the compressive halves of the respective autoencoders to the *D*-dimensional subspaces *Z*_1_ and *Z*_2_. These compressed subspaces were used to obtain reconstructed versions of *X*_1_ and *X*_2_, respectively denoted ${\hat{X}}_{1}$ and ${\hat{X}}_{2}$, using the expansive halves of the corresponding autoencoders. The cost function *C* used to train the Joint Autoencoder network not only minimized the reconstruction error for *X*_1_ and *X*_2_, but also the difference between *Z*_1_ and *Z*_2_:

$$C=MSE\;(X_{1},{\hat{X}}_{1})+MSE\;(X_{2},{\hat{X}}_{2})+MSE\;(Z_{1},Z_{2})$$ (Eq 3)

This design assumes that each of the partitions *X*_1_ and *X*_2_ contains the information necessary to robustly identify the underlying *D*-dimensional signals *Z*_1_ and *Z*_2_, but not the independent noise components that will differ between the two partitions. We trained the Joint Autoencoder using the ADAM optimizer with a learning rate *η* = 0.001 and dropout regularization on the input layer with *p* = 0.05. The use of Rectified Linear Unit (ReLU) activation functions in all layers ensured that the autoencoder network would both operate on and output non-negative signals while allowing for nonlinear embeddings. Our choice of using the ReLU activation function was motivated by its documented success in modeling a wide variety of nonlinearities for deep learning applications [56,57]. In addition, the strict nonnegativity of the ReLU function mimics that of real neural recordings.

### Statistical analyses

We used Monte Carlo simulations to generate up to 10 replications of synthetic data sets, each corresponding to multi-electrode array recording data from an experimental session. We noted the number of replications (n) in the figure captions where applicable. Our choice of the number of replications is reasonable compared to the number of experimental sessions that we would expect to see in experiments with monkeys [41,58,59]. The simulations differed by their random number generator seed, which dictated the pseudorandom sampling procedures required for generating the signals. There were three sampling steps in our simulations **(Fig 1)**. First was the creation of the low-dimensional latent signals, which were sampled from an empirical firing rate distribution. The second was the entries of the mixing matrix *W*, which were sampled from a zero-mean Gaussian distribution with unit variance. The third was the additive noise, sampled from a zero-mean Gaussian distribution with variance determined by the specified signal-to-noise ratio. We used bootstrapping with 10,000 iterations to compute the statistic of interest and computed its confidence interval using α = 0.05. We used Bonferroni correction for multiple comparisons.

## Results

Despite the large number of available algorithms for dimensionality estimation, there has been no systematic study of how well-suited they are for the analysis of neural data. Here we test several representative algorithms on synthetic datasets for which the intrinsic dimensionality is known, to assess their ability to estimate the true dimensionality of the data across a range of simulated conditions relevant to neuroscience. These assessments resulted in a recommended procedural pipeline for estimating the intrinsic dimensionality of a set of neural recordings.

### Dimensionality of noise-free datasets

**Fig 3:**
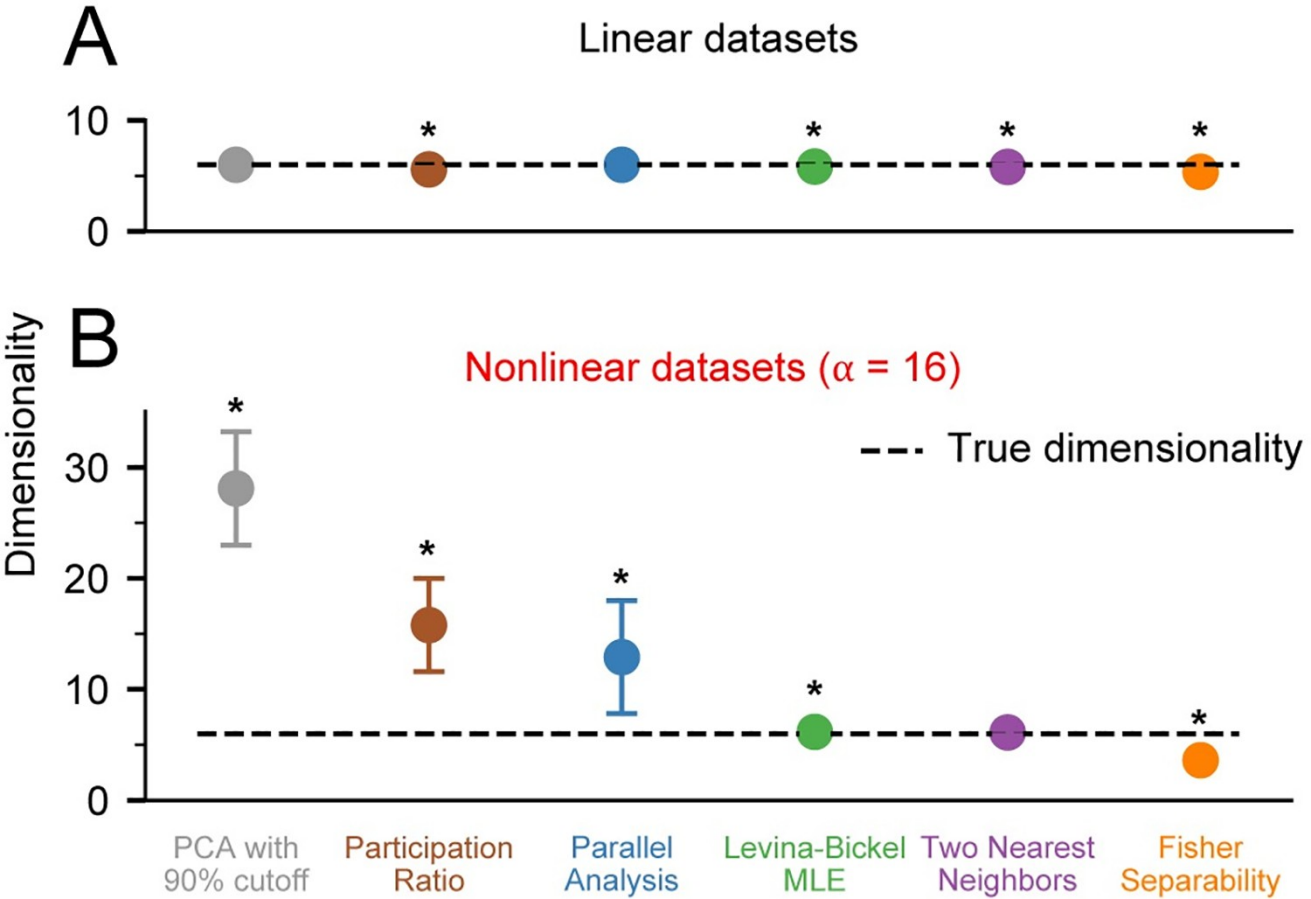
Dimensionality of noise free datasets. A) We applied PCA with 90% variance cutoff (PCA90, gray), Participation Ratio (PR, brown), Parallel Analysis (PA, blue), Levina-Bickel Maximum Likelihood Estimation (LBMLE, green), Two Nearest Neighbors (TNN, purple), and Fisher Separability Analysis (FSA, orange) to linearly embedded, *d* = 6 datasets (n = 10). B) Same as in A, but for nonlinearly embedded datasets. Circles indicate the mean and error bars indicate the standard deviation of the dimensionality estimates. Asterisks indicate significant difference of the mean from the true dimensionality of 6 at the significance level of α = 0.05.

We first considered the simplest case: how accurately can we determine the dimensionality of linearly embedded, noise-free datasets? To answer this question, we applied the six selected algorithms to datasets with dimensionality *d* = 6. We focused on *d* = 6 as this was the dimensionality estimate of actual multi-electrode array recordings found when using the methods investigated here. In this scenario, all tested linear and nonlinear algorithms estimated the true dimensionality accurately **(Fig 3A)**. Under noise-free conditions, the nonlinear algorithms were as accurate as the linear ones on linearly embedded datasets.

Next, we evaluated all algorithms on nonlinearly embedded noise-free datasets, also for *d* = 6. Nonlinearities were introduced as in Eq 1, using α = 16. In this case, the three linear algorithms dramatically overestimated the true dimensionality, with errors reaching more than 400% of the true value **(Fig 3B)**. In contrast, the nonlinear algorithms performed well; the Levina-Bickel Maximum Likelihood Estimation and the Two Nearest Neighbors methods were more accurate than Fisher Separability Analysis, which slightly underestimated the true dimensionality.

### Effect of non-uniform variances across channels

**Fig 4:**
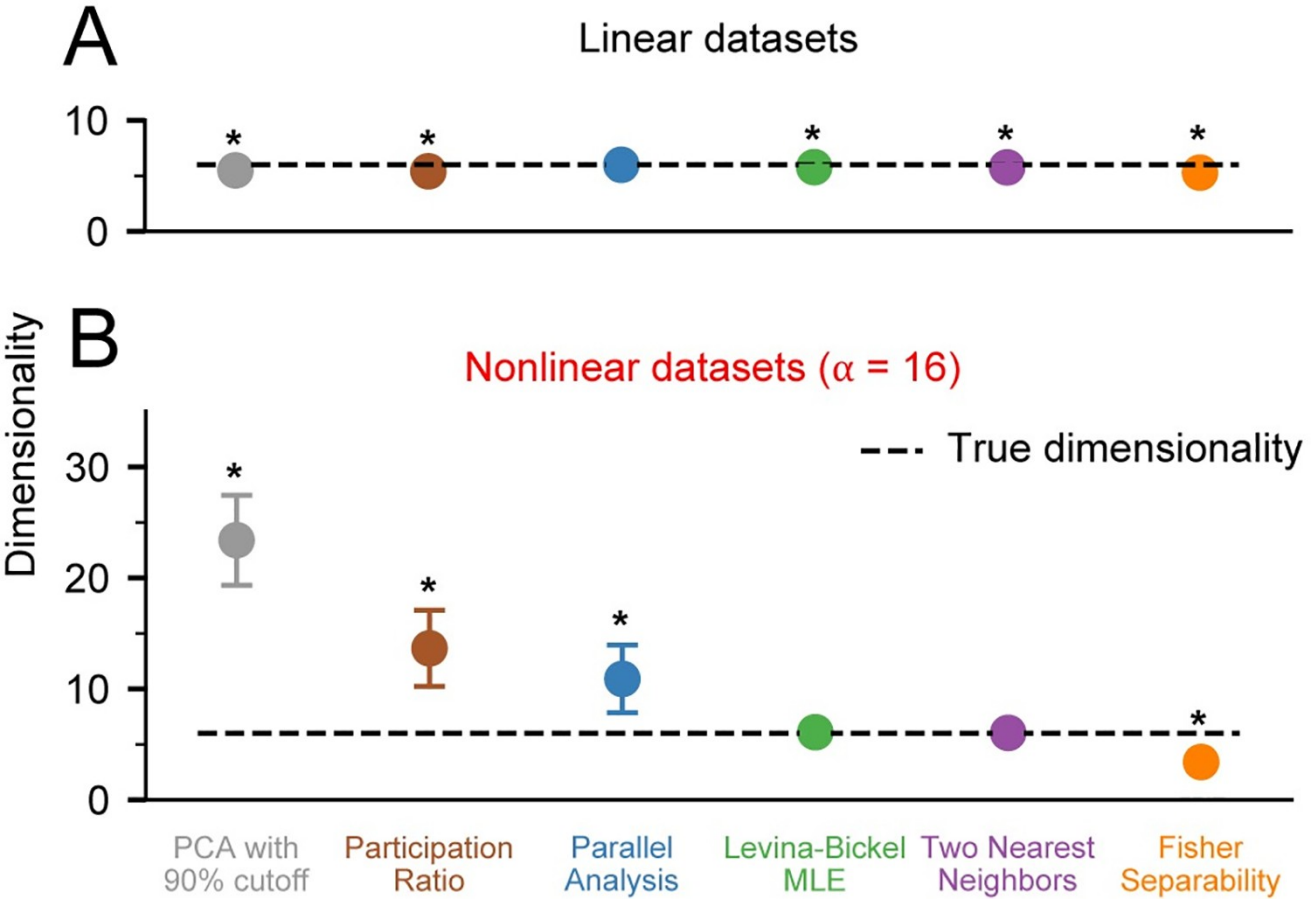
Dimensionality of noise free datasets with unequal variance. A) We applied PCA with 90% variance cutoff (PCA90, gray), Participation Ratio (PR, brown), Parallel Analysis (PA, blue), Levina-Bickel Maximum Likelihood Estimation (LBMLE, green), Two Nearest Neighbors (TNN, purple), and Fisher Separability Analysis (FSA, orange) to linearly embedded, *d* = 6 datasets (n = 10) after randomly scaling each of the *N* = 96 channels. B) Same as in A, but for nonlinearly embedded datasets. Circles indicate the mean and error bars indicate the standard deviation of the dimensionality estimates. Asterisks indicate significant differences of the mean from the true dimensionality of 6 at the significance level of α = 0.05.

The normalization of our simulated datasets restricted channel activity to the [0,1] interval, thus imposing a large degree of variance similarity across channels. In contrast, variances of real neural recordings can vary as much as 10-fold from channel to channel. To evaluate the performance of the dimensionality estimation algorithms considered here in the presence of non-uniform variances across channels, we scaled each channel of simulated neural data by a randomly chosen real number between 1 and 10. We found that most algorithms yielded lower estimates of dimensionality when applied to the rescaled data in comparison to the estimates obtained when the algorithms were applied to the data before rescaling **(Fig 4A and 4B)**. However, note that both Levina-Bickel Maximum Likelihood Estimation and the Two Nearest Neighbors yielded remarkably accurate dimensionality estimates when applied to rescaled data.

Because of the superior accuracy of Levina-Bickel Maximum Likelihood Estimation and Two Nearest Neighbors, we focused on these two methods for the remainder of the nonlinear analyses. We also retained Parallel Analysis as a benchmark for some of the analyses, as it was the most accurate linear method for estimating the dimensionality of nonlinearly embedded data.

### Effect of true dimensionality on algorithm accuracy

**Fig 5:**
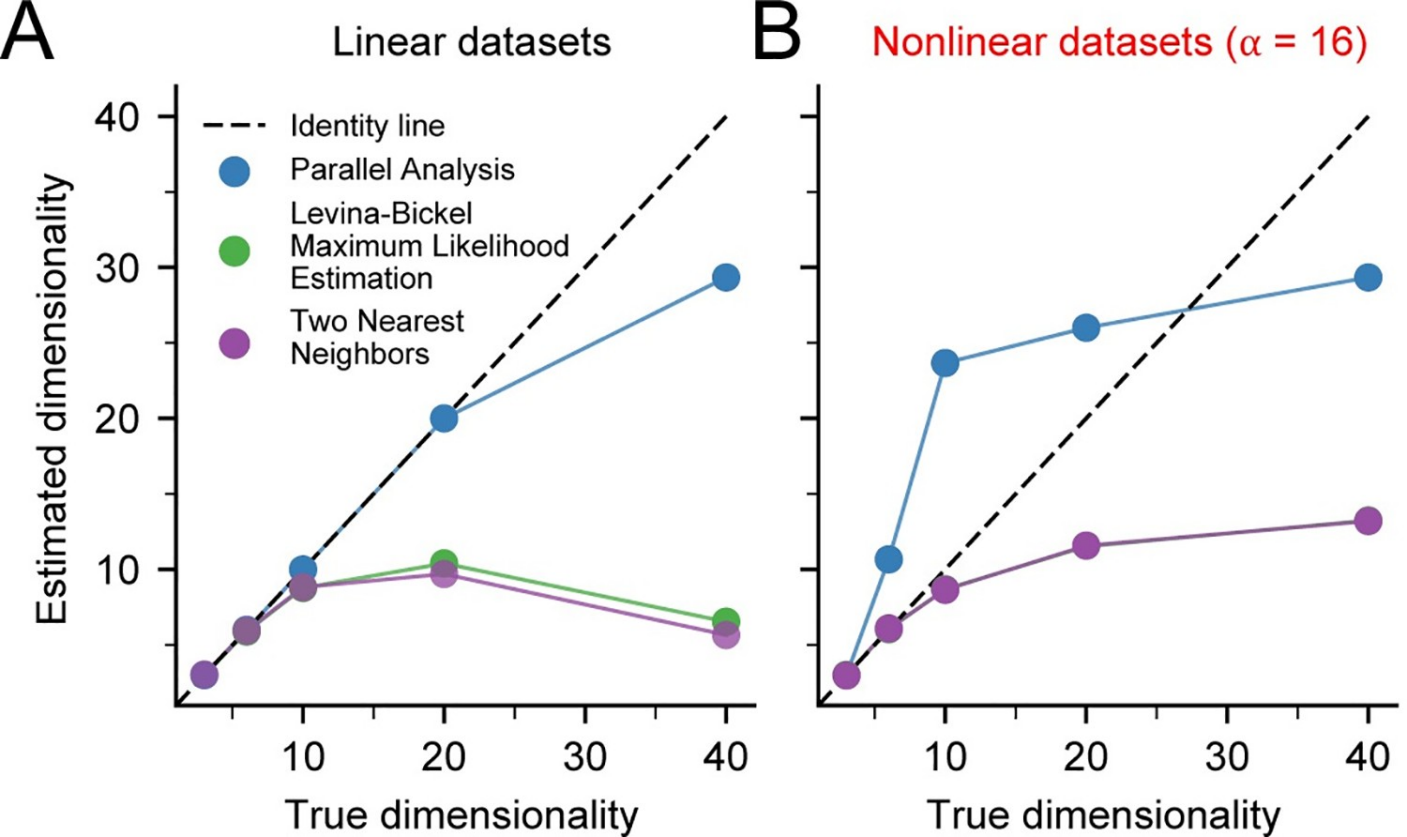
Effect of increasing true dimensionality on dimensionality estimates. A) The dimensionality of noise free, linear datasets (n = 3) was assessed using Parallel Analysis (PA), Levina-Bickel Maximum Likelihood Estimation (LBMLE), and Two Nearest Neighbors (TNN). Dashed line indicates the identity line. B) Same as A, but for nonlinear datasets. The curve for TNN precisely overlays that of LBMLE, causing it to be obscured.

We next evaluated how the true intrinsic dimensionality of the noise-free data influenced algorithm accuracy. Can any intrinsic dimensionality be reliably estimated? We found that the answer is no: the accuracy of all algorithms suffered when the intrinsic dimensionality of the synthetic data was too high. Parallel Analysis was accurate on linear datasets with *d* < 20, but inaccurate on nonlinear datasets of all dimensions, as expected **(Fig 5)**. Below about *d* = 6, Levina-Bickel Maximum Likelihood Estimation and Two-Nearest Neighbors were accurate on both linear and nonlinear datasets. However, Levina-Bickel Maximum Likelihood Estimation began to underestimate the dimensionality of both linearly embedded **(Fig 5A)** and nonlinearly embedded **(Fig 5B)** datasets for *d* > 6. This underestimation increased with increasing *d*. For nonlinear datasets, the estimate saturated at *d* = 13, where underestimation began to get much worse. These results revealed that the intrinsic dimensionality of nonlinearly embedded datasets is hard to estimate reliably when it is large.

### Effect of the level of nonlinearity

**Fig 6:**
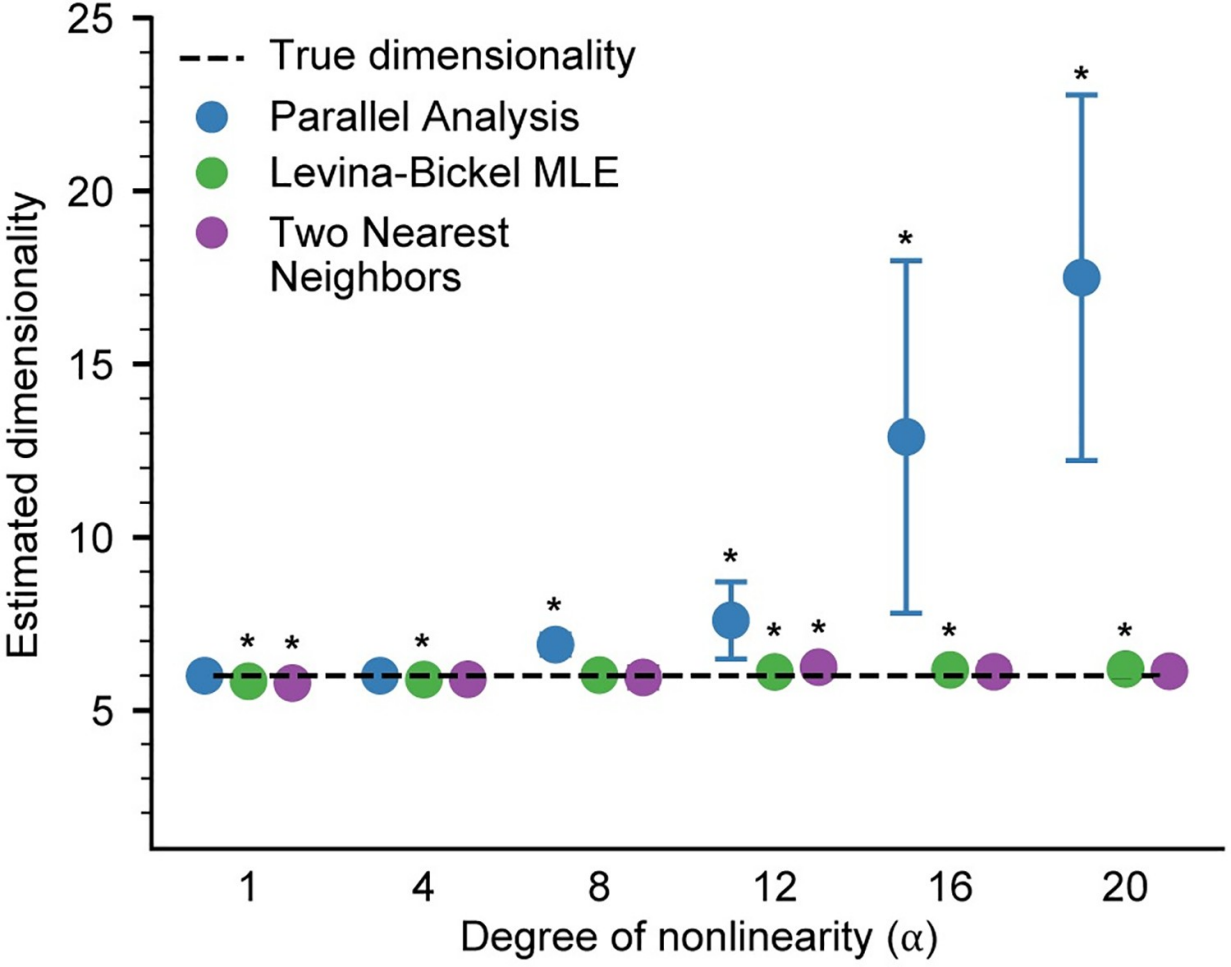
Effect of changing the degree of nonlinearity. Dimensionality of nonlinear datasets (n = 10) with varying levels of nonlinearity controlled by the α parameter (See Methods), was assessed using Parallel Analysis (PA), Levina-Bickel Maximum Likelihood Estimation (LBMLE), and Two Nearest Neighbors (TNN). Circles indicate the mean and error bars indicate the standard deviation of the dimensionality estimates. Asterisks indicate significant differences of the mean from the true dimensionality of 6 at the significance level of α = 0.05.

We next evaluated how the degree of nonlinearity influenced the accuracy of the dimensionality estimation algorithms. We controlled the degree of nonlinearity by varying the parameter *α* in Eq 1; this parameter controls the slope of the exponential activation function used to generate the nonlinearly embedded datasets. We found that both Levina-Bickel Maximum Likelihood Estimation and Two Nearest Neighbors provided accurate dimensionality estimates for all tested levels of nonlinearity (**Fig 6**). Surprisingly, even Parallel Analysis was accurate up to levels of nonlinearity corresponding to *α*≈8, where it started to overestimate the intrinsic dimensionality. These results revealed that Levina-Bickel Maximum Likelihood Estimation and Two Nearest Neighbors provide accurate dimensionality estimates for wide levels of nonlinearity, whereas Parallel Analysis is accurate only for low levels of nonlinearity.

### Amount of data required for estimating dimensionality

Ideally, algorithms would require only small amounts of data, so that the intrinsic dimensionality could be estimated even during transient behaviors and for a small number of recording channels. We thus evaluated the amount of data required to estimate the dimensionality of datasets with *d* = 6, by varying both the number of samples *M* and the number of recording channels *N*.

**Fig 7:**
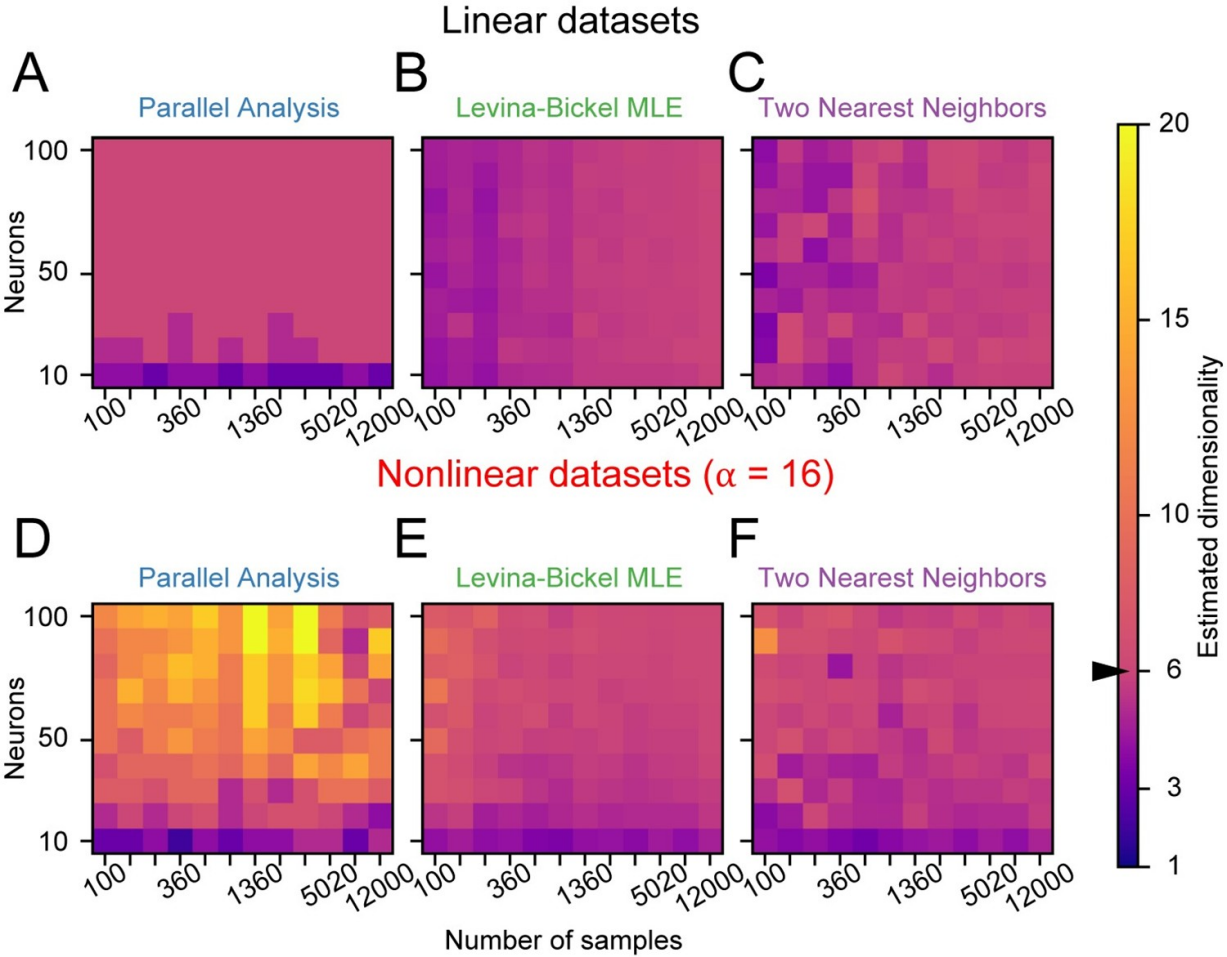
Amount of data required by dimensionality estimators. Amount of data required by A) Parallel Analysis (PA), B) Levina-Bickel Maximum Likelihood Estimation (LBMLE), and C) Two Nearest Neighbors (TNN) on linear datasets. Data length was logarithmically scaled between *M* = 100 and *M* = 12,000 samples. The correct dimensionality *d* = 6 is shown in pink. Light colors indicate overestimation and dark colors indicate underestimation of dimensionality. D, E, and F) Same as A, B, and C, respectively, but for nonlinear datasets.

On linear datasets, the accuracy of Parallel Analysis depended only on the number of channels: the algorithm was accurate if 20 or more channels were available (**Fig 7A**). In contrast, the accuracy of both Levina-Bickel Maximum Likelihood Estimation and Two Nearest Neighbors also depended on the number of samples (**Fig 7B and 7C**). Around *M* = 600, requiring about 30 seconds of data binned at 50 ms, was sufficient for accurate estimates of intrinsic dimensionality using either of these two nonlinear methods.

As expected for highly nonlinear datasets (*α* = 16, *d* = 6), Parallel Analysis was not accurate (**Fig 7D**) regardless of the amount of data. Both Levina-Bickel Maximum Likelihood Estimation and Two Nearest Neighbors were accurate provided that data from more than 50 channels were available (**Fig 7E and 7F**). Furthermore, while Levina-Bickel Maximum Likelihood estimation required around 600 samples of data for accurate dimensionality estimates, Two Nearest Neighbors required more than twice as many samples. These results would also depend on the actual dimensionality *d*; here we focused on *d =* 6.

### Evaluating and reducing the effects of noise

**Fig 8:**
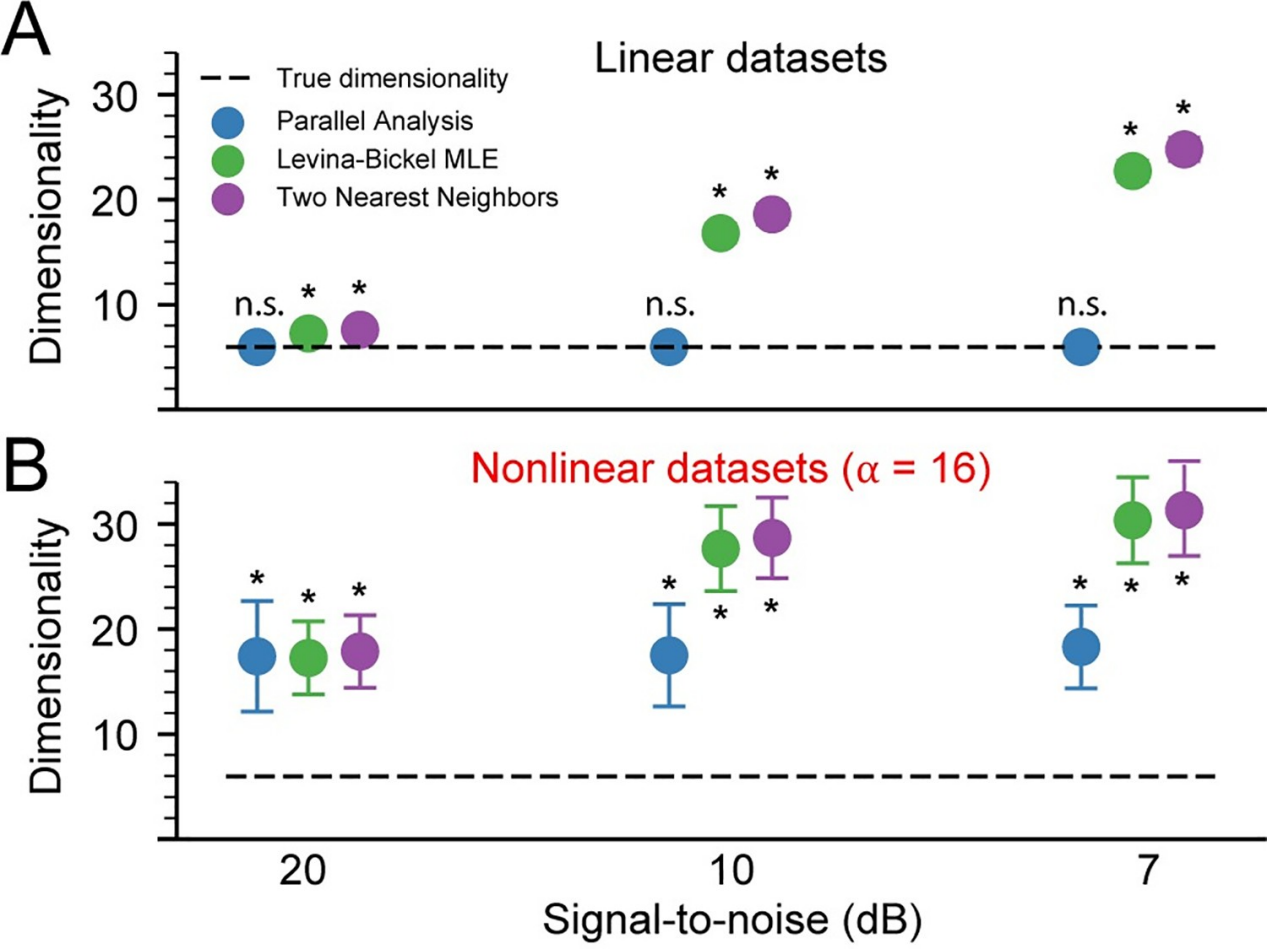
Effect of noise on dimensionality estimates. Estimated dimensionality of linear (A) and nonlinear (B) datasets (n = 10) with 20 dB, 10 dB, and 7 dB signal-to-noise ratio was assessed using Parallel Analysis (PA), Levina-Bickel Maximum Likelihood Estimation (LBMLE), and Two Nearest Neighbors (TNN). Circles indicate the mean and error bars indicate the standard deviation of the dimensionality estimates. Asterisks indicate significant differences of the mean from the true dimensionality of 6 at the significance level of α = 0.05.

Any experiment will include some amount of noise in the recorded signals. As expected, all tested algorithms overestimated intrinsic dimensionality in the presence of noise (**Fig 8**). For any given noise level, estimation errors for the linear datasets **(Fig 8A)** were a bit smaller than those for the nonlinear datasets **(Fig 8B)**. Adding noise with a power of only 1% of that of the signal (SNR = 20 dB) caused Levina-Bickel Maximum Likelihood Estimation and Two Nearest Neighbors to overestimate the dimensionality of the nonlinear data by ~200% **(Fig 8B)**. PA yielded consistent overestimation errors across all nonzero levels of noise for both linear and nonlinear data.

**Fig 9:**
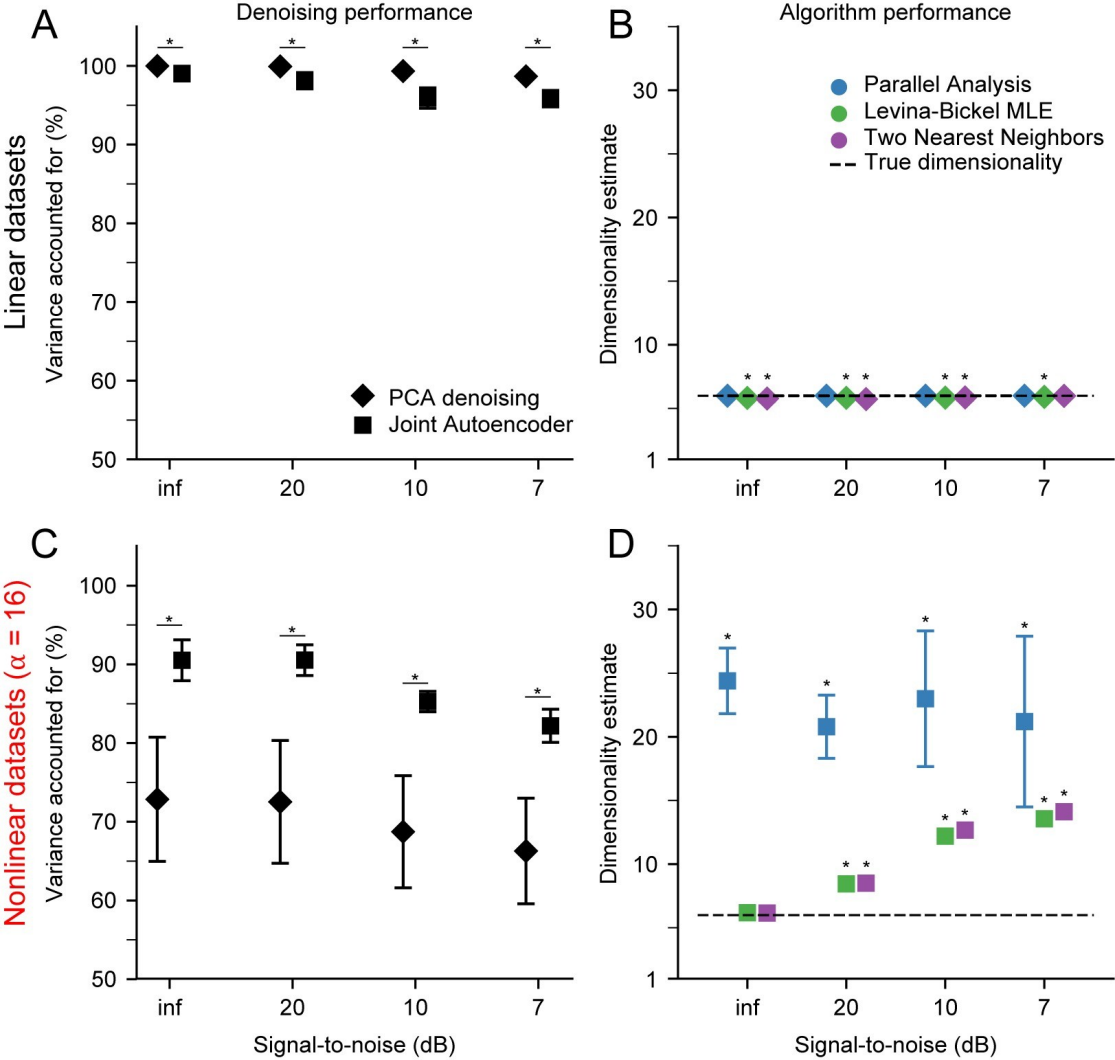
Performance of PCA and Joint Autoencoder (JAE) denoising algorithms. A) PCA and JAE denoising applied to linear datasets (n = 10) with varying signal-to-noise ratio. Symbols indicate the mean and error bars indicate the standard deviation of the VAF between noise-free and denoised signals. Asterisks indicate significant difference between mean values at the significance level of α = 0.05. B) Dimensionality estimation on linear datasets after PCA denoising. Dimensionality was estimated using Parallel Analysis (PA), Levina-Bickel Maximum Likelihood Estimation (LBMLE), and Two Nearest Neighbors (TNN). Symbols indicate the mean and error bars indicate the standard deviation of the dimensionality estimates. Asterisks indicate significant differences of the mean from the true dimensionality of 6 at the significance level of α = 0.05. C) Same as in A, but for nonlinear datasets. D) Same as in B, but for nonlinear datasets after JAE denoising.

We evaluated two algorithms for mitigating the effects of noise prior to estimating dimensionality: a PCA-based linear method and a Joint Autoencoder nonlinear neural network (see Methods). Both methods were quite effective for denoising the linear datasets **(Fig 9A)**, with the PCA-based approach slightly better than the Joint Autoencoder at the higher noise levels. For linear datasets, dimensionality estimates following PCA-based denoising were highly accurate, yielding correct estimates of the true intrinsic dimension even for high-noise signals **(Fig 9B)**. The Joint Autoencoder was significantly more effective for denoising the nonlinear datasets **(Fig 9C)**. Joint Autoencoder denoising on nonlinear datasets resulted in dimensionality estimates that still increasingly overestimated with increasing noise, but at a much slower rate than without denoising **(Fig 9D)**. The highest noise level we tested (20%; SNR = 7 dB) caused the dimensionality to be overestimated by about 100%. These results were consistent for different degrees of nonlinearity. The more nonlinear the data, the more appropriate it was to use the Joint Autoencoder for denoising.

## Discussion

This study evaluated techniques for estimating the intrinsic dimensionality of high-dimensional neural recordings. We considered representative linear and nonlinear algorithms, testing their performance on synthetic datasets that captured properties of neural recordings likely to affect dimensionality estimation. The tested datasets had known intrinsic dimensionality, known levels of noise, and embeddings that were either linear or nonlinear. Our results demonstrated that none of the tested algorithms work for all possible scenarios, but they yielded important insights for when estimates of intrinsic dimensionality are likely to be valid and when they are not. As expected, we found that linear estimation methods are generally not as accurate as nonlinear methods when the mapping between the low-dimensional latent space and the high-dimensional space of neural recordings is nonlinear. Surprisingly, the linear method Parallel Analysis estimated the dimensionality of mildly nonlinear datasets well though it failed for more highly nonlinear embeddings. In contrast, the nonlinear methods worked well on both linear and highly nonlinear datasets but failed once the intrinsic dimensionality of the data became too high.

Noise was a challenge for all methods, causing dimensionality to be overestimated even for signal-to-noise ratios as low as 20 dB (1% noise variance). We presented two approaches for denoising the data so as to improve the accuracy of the dimensionality estimation. These were a linear PCA-based approach and a novel nonlinear, deep learning approach that we call the Joint Autoencoder. Both denoising approaches attempted to remove signal components that were not shared across the data channels. To achieve this, the PCA-based approach simply removed Principal Components with low variance, whereas the Joint Autoencoder identified an underlying manifold that was common to two randomly sampled sets of channels. Both approaches relied on a linear, upper-bound estimate of the intrinsic dimensionality. Denoising by either method substantially improved subsequent dimensionality estimation, but the Joint Autoencoder was substantially more effective in denoising nonlinear datasets. For linear datasets, dimensionality estimates using Parallel Analysis, Levina-Bickel Maximum Likelihood Estimation, and Two Nearest Neighbors were accurate after PCA-denoising. In the nonlinear case, dimensionality estimates using the two nonlinear methods (LBMLE and TNN) were similarly accurate after JAE-denoising.

### Implications for evaluation of experimental recordings

Due to its computational efficiency and ease of interpretation, most studies have used PCA with an arbitrary variance cutoff to estimate the dimensionality of M1 neural recordings [4,17,41,44,45]. While we have shown that some of the linear methods can be quite effective, simply eliminating non leading PCs based on a cumulative variance cutoff was the least accurate of the algorithms that we tested. Parallel Analysis, the most accurate linear method, performed as well or even better than some of the more advanced and computationally demanding nonlinear methods. Therefore, PA should suffice as a quick and effective approach to estimating dimensionality, even for mildly noisy and nonlinear datasets.

Some of the linear methods used in neuroscience studies rely on a structure of repeated trials in the data [7,46]. These methods use the regularity of repeated trials in a supervised scenario to identify neural dimensions associated with specific experimentally controlled conditions. Such supervised methods cannot be applied to data obtained during non-stereotyped, non-repeating behaviors. All of the methods that we assess in this study are unsupervised and thus applicable to datasets with no repeated trial structure.

Despite the simplicity of linear algorithms, estimating the dimensionality of nonlinear manifolds requires nonlinear algorithms. There is some evidence that neural manifolds may be nonlinear. Recent studies have shown that nonlinear methods for inferring behavioral parameters from M1 neural manifolds are superior to linear methods [60–63]. This suggests that the underlying neural manifold representing motor intent may be nonlinear, and that linear dimensionality estimation methods may be inadequate when estimating the intrinsic dimensionality of primary motor cortical recordings. Studies that investigated the dimensionality of M1 using linear methods most likely overestimated its true intrinsic dimensionality.

Nonlinear algorithms were more accurate than linear algorithms for nonlinear datasets of dimensionality below 10. However, nonlinear methods underestimated dimensionalities above 10. This is a critical concern for experimental recordings, since a low dimensionality estimate from a nonlinear method might be inaccurate if the true dimensionality were large. Multiple studies using linear methods have reported an estimated dimensionality of M1 of around 10 for simple, well-practiced behaviors [5,45,59]. Our results show that linear methods provide an upper bound to the estimate of intrinsic dimensionality as long as the true dimensionality of the data is below 20. If the intrinsic dimensionality of M1 is substantially higher for more dexterous use of arm and hand than for the scenarios that have typically been studied, the nonlinear methods investigated here may underestimate it.

One method for addressing this concern would be to use nonlinear methods to reduce the dimensionality of a dataset to that of its nonlinear dimensionality estimate, and then to assess the amount of variance that the nonlinear low-dimensional representation captures. If the VAF is high, the data may be truly nonlinearly low dimensional. If, on the other hand, the VAF is low, the true intrinsic dimensionality could be higher than estimated. For the latter case, a practical approach would be to report only the linear dimensionality estimate and emphasize that it only provides an upper bound to the true dimensionality.

We currently lack techniques for reliably assessing datasets with high intrinsic dimensionality, at least when considering practical situations with limited data. There have been some theoretical studies of the amount of data needed for accurate estimation of dimensionality [64,65]. Correlation Dimension, the method on which many nonlinear algorithms are based, requires that the number of data samples *M* be on the order of 10^d/2^ [29]. The total amount of data can be increased by either recording from more channels or for a longer duration. Studies that investigated the dimensionality of the primary visual cortex (V1) found that the eigenvalue spectrum of the neural signals obtained from approximately one thousand neurons decayed as a power law [66,67]. These findings would not have been possible if recording from a hundred neurons, which would not have revealed the long, slow-decaying tail of the eigenvalue distribution. One interpretation of these findings is that the linear dimensionality of V1 is arbitrarily large. However, an alternative interpretation is that the neural data are embedded in a very nonlinear manifold, causing the intrinsic dimensionality to be overestimated by the linear methods used in these studies.

The stochastic nature of neural firing and the noise associated with experimental measurements will also cause the intrinsic dimensionality to be overestimated. The two denoising approaches that we presented are simple and effective. Depending on the assumptions about the underlying structure of firing patterns, alternative denoising approaches may be useful. For example, if the temporal relationship between the firing patterns of the population neural activity is of interest, one could use denoising methods that explicitly attempt to model these dynamics, such as Latent Factor Analysis through Dynamical Systems (LFADS), prior to estimating the dimensionality [61].

For the past five decades since the time of Evarts’ early experiments [68], assessing the relationship between behaviors and single neuron signals recorded from the brain has been a mainstay of motor systems research. Although the focus of our study was on the dimensionality of the neural manifold to which the population activity is confined, the natural next step in the analysis is to investigate the dynamics of the signals within the neural manifold and their relation to behavior.

### Limitations of the study

While we tried to replicate essential features of experimental data, there are certain characteristics that we did not try to model in our simulations. For example, we only considered additive Gaussian isotropic noise, for simplicity. Experimental recordings might include non-additive, non-isotropic, or non-Gaussian noise. In such cases, PCA may not be an appropriate approach to denoising, even for linearly embedded data. Methods such as Factor Analysis or extensions such as Gaussian-Process Factor Analysis [69], and preprocessing steps such as square-root transforms or pre-whitening could be used instead.

We scaled the firing rates of each channel to be in the [0,1] range. The arbitrary scaling of firing rates provided a simple means for the nonlinear datasets to have the same range as their linear counterparts, as the nonlinear activation function that we used mapped the [0,1] range onto itself. However, this modeling restriction does not reflect experimental neural firing data, since the range of neural firing can differ significantly even across neurons of the same type. We have illustrated how heterogeneity in the range of firing rates affects the reliability of dimension estimation algorithms. Soft-normalization approaches that are commonly used in neuroscience (dividing a neuron’s firing rate by its range plus a small constant, e.g. [70]), would results in the amplification of signals with low variance and would cause variance-based algorithms to result in higher dimensionality estimates.

The latent signals used to generate simulated firing rates have the same first-order statistics as the actual data from which they were sampled, but the rescaling of simulated channel activity to a [0,1] range introduced a departure from realism. This unrealistic scenario is addressed through the random rescaling of individual simulated channels, to reflect the heterogeneity in the range of firing rates observed in actual neural recordings. The latent signals corresponding to the rescaled data no longer share common statistics. This scenario allows us to address an important problem: that low-variance latent signals can be informative [71]. As demonstrated in our study, the use of nonlinear methods for dimensionality estimation ameliorates the problems that arise when neglecting low-variance signals as purely noisy.

### Recommended analysis pipeline

**Fig 10:**
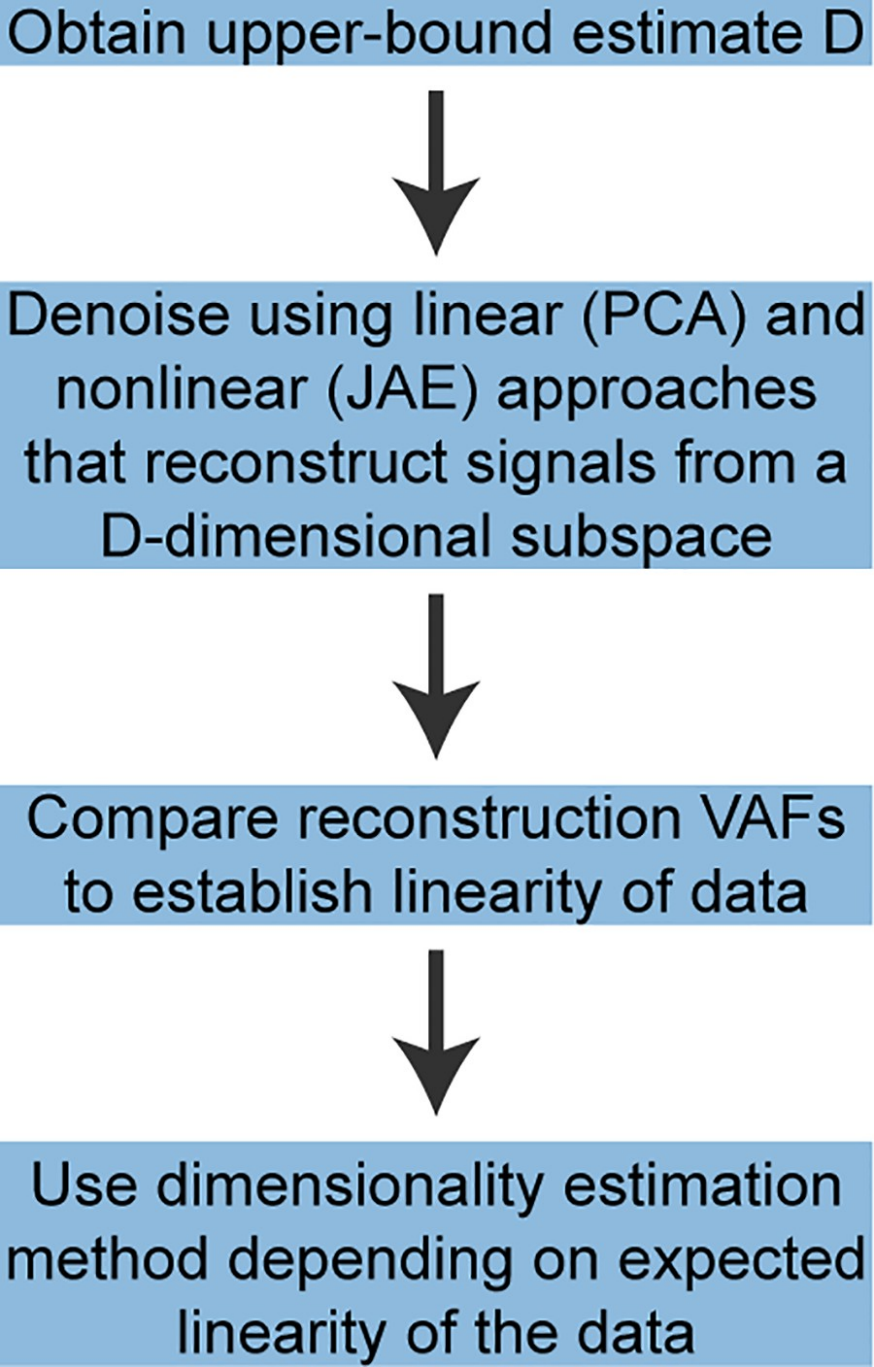
Recommended analysis pipeline for estimating the dimensionality of multi-electrode array recordings. First, obtain an upper-bound dimensionality estimate *D* using a linear algorithm. Parallel Analysis works well for this purpose. Next, denoise the data using both linear (PCA based) and nonlinear (JAE based) denoising approaches and compare their reconstruction VAFs. Higher VAF for the PCA based denoising. Similar VAFs for the linear and nonlinear denoising approaches would signal a linear manifold. In contrast, higher VAF for the JAE based denoising would signal a nonlinear manifold. Finally, once the signals have been denoised using the appropriate denoising method based on the determined linearity of the manifold, estimate the dimensionality of the denoised signals. Parallel Analysis is appropriate for linear manifolds. Levina-Bickel Maximum Likelihood Estimation and Two Nearest Neighbor are the most accurate nonlinear algorithms that we tested.

Based on our results, we recommend the following approach for estimating the dimensionality of neural recordings **(Fig 10)**. First, obtain an upper-bound estimate *D* of the intrinsic dimensionality of the data. We found that Parallel Analysis works well for this purpose, being both computationally efficient and the most accurate linear method in our tests. Next, the signals should be denoised. Our denoising approach worked by projecting the neural signals into a subspace of dimensionality *D* equal to the upper-bound dimensionality estimate, and then reconstructing them based on these projections. A PCA based reconstruction is easy to implement and interpret and may be preferable if computational efficiency is important. A nonlinear denoising algorithm, such as the Joint Autoencoder we proposed, should also be used to assess the degree of nonlinearity of the manifold. The usefulness of the denoising step was quantified through the VAF between the reconstructed signals, assumed to be denoised, and the noise-free synthetic signals before noise was added to them. Our results showed that for nonlinear datasets this VAF was higher for the Joint Autoencoder than it was for PCA. However, this VAF cannot be computed for experimental data, for which we do not have access to the noise-free signals. In this scenario, the reconstruction VAF between noisy inputs and the denoised reconstructed outputs may be useful for detecting nonlinear manifolds: a higher reconstruction VAF for Joint Autoencoder denoising than for PCA denoising would signal a nonlinear manifold. A reconstruction VAF that prefers the Joint Autoencoder indicates that this denoising method yields better denoised signals. Once the signals are denoised, and the linearity or nonlinearity of the manifold is established, either a linear or nonlinear dimensionality estimation method should be used depending on the comparative performance of the corresponding denoising algorithms. The most accurate linear method we tested was Parallel Analysis. Of the nonlinear methods, Levina-Bickel Maximum Likelihood Estimation and Two Nearest Neighbors were the most accurate; Levina-Bickel Maximum Likelihood Estimation required fewer data samples.

### Conclusions

Estimating the dimensionality of neural data is challenging. In this study, we tested several available algorithms and determined the conditions under which estimating dimensionality may be particularly difficult or even impractical. Noise is a confounding factor and must be removed prior to dimensionality estimation. Most existing studies have estimated intrinsic dimensionality using linear methods that are computationally efficient and easy to interpret. We showed that linear methods provide an upper-bound to the intrinsic dimensionality, and in cases of high noise, may even provide better estimates than nonlinear methods, although neither linear nor nonlinear methods will yield accurate estimates in this scenario. Nonlinear algorithms were more accurate for nonlinear datasets when noise was adequately removed. Finally, algorithms failed when the intrinsic dimensionality was high. It may be impractical or impossible to estimate the dimensionality of neural data when it is above ~20. However, estimation of the dimensionality of neural activity in the primary motor cortex may be possible, as many studies have reported its linear dimensionality to be within the practical limits for accurate estimation by the methods we tested.

## Supporting information

S1 Table Application of the recommended analysis pipeline to three sets of real neural recordings. The parallel analysis (PA) estimates of the dimensionality are shown for each of the datasets J1, J2, and J3. These values determined the dimensionality to be used for denoising each dataset. The PCA-based denoising yielded reconstructions with 53%, 51%, and 56% VAF. The JAE-based denoising was slightly better for all datasets, with 61%, 59%, and 62% VAF. The better performance of the JAE-based denoising is indicative of modest nonlinearity in all three datasets. Once each dataset had been denoised using JAE, the corresponding dimensionalities were estimated using MLE and TNN. These results motivated our choice of *d* = 6 for the intrinsic dimensionality of most of our simulated datasets.
(DOCX)
