# Supplementary material for: Estimating the dimensionality of the manifold underlying multi-electrode neural recordings
Source: PLoS Comput Biol. 2021 Nov 29;17(11):e1008591. doi: 10.1371/journal.pcbi.1008591 (PMC8659648; doi:10.1371/journal.pcbi.1008591)
Supplement: S1 Table — The parallel analysis (PA) estimates of the dimensionality are shown for each of the datasets J1, J2, and J3. These values determined the dimensionality to be used for denoising each dataset. The PCA-based denoising yielded reconstructions with 53%, 51%, and 56% VAF. The JAE-based denoising was slightly better for all datasets, with 61%, 59%, and 62% VAF. The better performance of the JAE-based denoising is indicative of modest nonlinearity in all three datasets. Once each dataset had been denoised using JAE, the corresponding dimensionalities were estimated using MLE and TNN. These results motivated our choice of d = 6 for the intrinsic dimensionality of most of our simulated datasets. (DOCX) [file pcbi.1008591.s001.docx]

Supporting Information

In order to apply our recommended analysis pipeline to actual neural data, we used neural activity recorded from the primary motor cortex (M1) of a macaque monkey (Monkey J) during the execution of an isometric center-out task (see [1] for details). We used three datasets, J1, J2, and J3, corresponding to three different experimental sessions. The recorded neural data consisted of 96-channels of spiking activity, binned at 50 ms and smoothed using a Gaussian kernel with zero mean and a s.d of 50 ms to obtain firing rates. We selected five-minute segments of concatenated, successful trials taken from each of the three datasets **(S1 Table)**. This is the actual neural data to which we applied the recommended analysis pipeline.

The analysis pipeline, described in the main body of the paper (see “Recommended analysis pipeline” in “Discussion”), consists of three steps: 1) Obtaining the upper bound dimensionality, 2) denoising, and 3) estimating dimensionality. We chose Parallel Analysis (PA) for Step 1. The estimated upper bound dimensionalities are shown in the Table below. The next step was to denoise the datasets using PCA and JAE, by reconstructing the data after compression through a low-dimensional bottleneck. The VAF between the original and reconstructed data using JAE-based denoising was consistently higher than that obtained when using PCA-based denoising, signaling nonlinearity in all three datasets. The final step was to apply the two nonlinear dimensionality estimators, Levina-Bickel Maximum Likelihood (MLE) and Two Nearest Neighbors (TNN) to the denoised datasets. The MLE and TNN estimates are shown in the Table below. Based on these dimensionality estimates, we chose an intrinsic dimensionality $d$=6 for most of the simulated neural activity.

|  | **Step 1**  **Upper bound dimensionality** | **Step 2**  **VAF after linear and nonlinear denoising** | | **Step 3**  **Estimate**  **dimensionality** | |
| --- | --- | --- | --- | --- | --- |
| **Dataset** | **PA** | **PCA VAF** | **JAE VAF** | **MLE** | **TNN** |
| J1 | 11 | 53% | 61% | 6.8 | 5.8 |
| J2 | 11 | 51% | 59% | 6.7 | 5.5 |
| J3 | 9 | 56% | 62% | 4.8 | 4.6 |
|  |  |  |  |  |  |

**S1 Table: Application of the recommended analysis pipeline to three sets of real neural recordings**. The parallel analysis (PA) estimates of the dimensionality are shown for each of the datasets J1, J2, and J3. These values determined the bottleneck dimensionality to be used for denoising each dataset. The PCA-based denoising yielded reconstructions with 53%, 51%, and 56% VAF. The JAE-based denoising was slightly better for all datasets, with 61%, 59%, and 62% VAF. The better performance of the JAE-based denoising is indicative of modest nonlinearity in all three datasets. Once each dataset had been denoised using JAE, the corresponding dimensionalities were estimated using MLE and TNN. These results motivated our choice of $d$=6 for the intrinsic dimensionality of most of our simulated datasets.

**References**

1. Gallego JA, Perich MG, Naufel SN, Ethier C, Solla SA, Miller LE. Cortical population activity within a preserved neural manifold underlies multiple motor behaviors. Nat Commun. 2018;9(1):4233. Epub 2018/10/14. doi: 10.1038/s41467-018-06560-z. PubMed PMID: 30315158; PubMed Central PMCID: PMCPMC6185944.
